# Supplementary material for: Exploring spatial clusters of caesarean sections across India - Insights from National Family Health Survey data
Source: PLOS Glob Public Health. 2026 Mar 5;6(3):e0006070. doi: 10.1371/journal.pgph.0006070 (PMC12962522; doi:10.1371/journal.pgph.0006070)
Supplement: S1 Text — This document contains additional methods, tables (Table A & Table B), and figures (Fig A & Fig B). (DOCX) [file pgph.0006070.s001.docx]

Supplementary Appendix

Exploring Spatial Clusters of Caesarean Sections across India - Insights from NFHS Data

Table of contents

[1. Additional Methods 1](#_Toc196215908)

[Data Sources 1](#_Toc196215909)

[Spatial Analysis 2](#_Toc196215910)

[Coordinate Reference System (CRS) 2](#_Toc196215911)

[Spatial Data Preparation and Cleaning 2](#_Toc196215912)

[Spatial Weight Matrix 2](#_Toc196215913)

[Global Moran’s I 3](#_Toc196215914)

[Local Indicators of Spatial Association (LISA) 4](#_Toc196215915)

[Getis-Ord General G 4](#_Toc196215916)

[List of R Packages Used 5](#_Toc196215917)

[2. Additional Tables 6](#_Toc196215918)

[3. Additional Figures 8](#_Toc196215919)

# 1. Additional Methods

## Data Sources

This study draws on nationally representative secondary datasets and official shapefiles, as described below:

- **National Family Health Survey (NFHS)**
  The NFHS rounds 4 (2015–16) and 5 (2019–21) were used to obtain district-level indicators on maternal and child health, including cesarean section rates, institutional delivery, antenatal care coverage, and child nutritional status (stunting, wasting, underweight). District-wise fact sheets and as raw *.pdf files were accessed from the official [NFHS portal](http://rchiips.org/NFHS/).
- **Survey of India Administrative Boundaries**
  District- and state-level shapefiles were obtained from the Survey of India’s open map portal and used for spatial mapping and geocoding. When mismatches arose (e.g., due to administrative redistricting), state-level imputation was applied to maintain analytical consistency.

All non-spatial datasets used were publicly accessible, non-identifiable, and used in accordance with ethical norms for secondary data analysis. A harmonized, cleaned dataset was developed using R to allow spatial linkage and time-trend analyses.

## Spatial Analysis

### Coordinate Reference System (CRS)

All spatial data layers were projected to **EPSG:4326 (WGS84)** to ensure consistency in spatial referencing. This CRS is widely supported and appropriate for national-level analysis.

### Spatial Data Preparation and Cleaning

- NFHS district-level data were extracted from state and district PDF fact sheets using tabulizer::extract_tables().
- Administrative boundary shapefiles were obtained from the Survey of India’s open map portal.
- Shapefiles were simplified using rmapshaper::ms_simplify() for plotting efficiency.
- District name mismatches were resolved using fuzzy string matching (stringdist package).
- When district boundaries differed between NFHS rounds and shapefiles, values were imputed using state-level averages.

### Spatial Weight Matrix

A queen contiguity matrix was created using the rgeoda::queen_weights() function:

This spatial weights matrix forms the basis for calculating spatial autocorrelation.

###

### Global Moran’s I

Global spatial autocorrelation was assessed using Moran’s I:


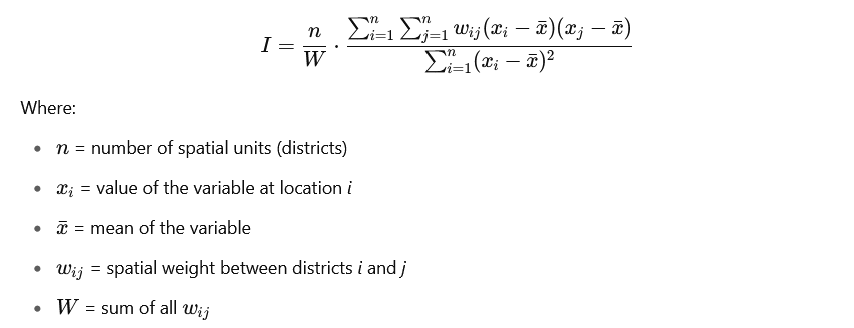


The null hypothesis assumes spatial randomness. A positive and significant Moran’s I indicates clustering.

### Local Indicators of Spatial Association (LISA)

Univariate LISA was used to detect local clusters and spatial outliers. The LISA statistic is calculated as:


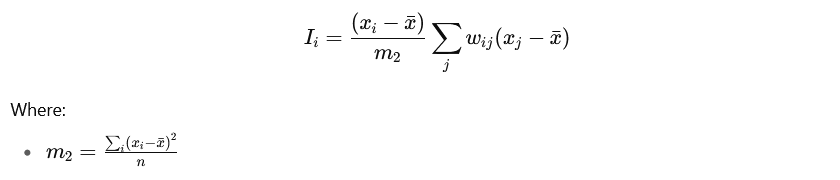


Cluster types: - **High-High**: Hotspots - **Low-Low**: Coldspots - **High-Low**, **Low-High**: Spatial outliers

Bivariate LISA was used to explore spatial correlations between pairs of variables (e.g., C-sections and institutional delivery).

### Getis-Ord General G

Hotspot detection was conducted using the Getis-Ord General G statistic:


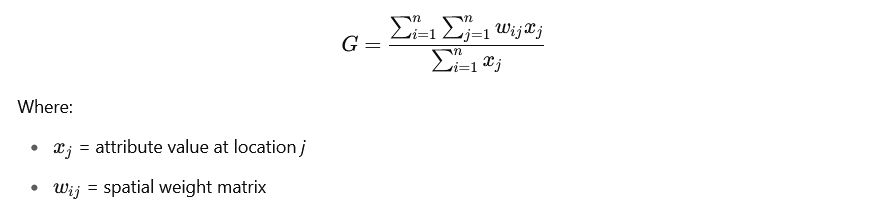


A high *z*-score suggests clustering of high values (hotspots), while a low *z*-score indicates clustering of low values (coldspots). The null hypothesis assumes no spatial clustering.

## List of R Packages Used

The R packages used in the study are as follows:

1. pak
2. here
3. fs
4. rvest
5. tidyverse
6. sf
7. patchwork
8. rgeoda
9. gstat
10. gt
11. gtExtras
12. gtsummary
13. rmapshaper
14. tabulapdf
15. pdftools
16. janitor

Csárdi G and Hester J (2024) *pak: Another Approach to Package Installation*. Available at: <https://CRAN.R-project.org/package=pak>.

Firke S (2023) *janitor: Simple Tools for Examining and Cleaning Dirty Data*. Available at: <https://CRAN.R-project.org/package=janitor>.

Gräler B, Pebesma E and Heuvelink G (2016) [Spatio-temporal interpolation using gstat](https://journal.r-project.org/archive/2016/RJ-2016-014/index.html). *The R Journal* 8: 204–218.

Hester J, Wickham H and Csárdi G (2024) *fs: Cross-Platform File System Operations Based on ‘libuv’*. Available at: <https://CRAN.R-project.org/package=fs>.

Iannone R, Cheng J, Schloerke B, et al. (2024) *gt: Easily Create Presentation-Ready Display Tables*. Available at: <https://CRAN.R-project.org/package=gt>.

Li X and Anselin L (2023) *rgeoda: R Library for Spatial Data Analysis*. Available at: <https://CRAN.R-project.org/package=rgeoda>.

Mock T (2023) *gtExtras: Extending ‘gt’ for Beautiful HTML Tables*. Available at: <https://CRAN.R-project.org/package=gtExtras>.

Müller K (2020) *here: A Simpler Way to Find Your Files*. Available at: <https://CRAN.R-project.org/package=here>.

Ooms J (2024) *pdftools: Text Extraction, Rendering and Converting of PDF Documents*. Available at: <https://CRAN.R-project.org/package=pdftools>.

Pebesma E (2018) [Simple Features for R: Standardized Support for Spatial Vector Data](https://doi.org/10.32614/RJ-2018-009). *The R Journal* 10(1): 439–446.

Pebesma E and Bivand R (2023) *Spatial Data Science: With applications in R*. Chapman and Hall/CRC. Available at: <https://r-spatial.org/book/>.

Pebesma EJ (2004) Multivariable geostatistics in S: The gstat package. *Computers & Geosciences* 30: 683–691.

Pedersen TL (2024) *patchwork: The Composer of Plots*. Available at: <https://CRAN.R-project.org/package=patchwork>.

Rodriguez-Sanchez F and Jackson CP (2024) *grateful: Facilitate Citation of R Packages*. Available at: <https://pakillo.github.io/grateful/>.

Sepulveda MV (2024) *tabulapdf: Extract Tables from PDF Documents*. Available at: <https://github.com/ropensci/tabulapdf>.

Sjoberg DD, Whiting K, Curry M, et al. (2021) [Reproducible summary tables with the gtsummary package](https://doi.org/10.32614/RJ-2021-053). *The R Journal* 13: 570–580.

Teucher A and Russell K (2023) *rmapshaper: Client for ‘mapshaper’ for ‘Geospatial’ Operations*. Available at: <https://CRAN.R-project.org/package=rmapshaper>.

Wickham H, Averick M, Bryan J, et al. (2019) [Welcome to the tidyverse](https://doi.org/10.21105/joss.01686). *Journal of Open Source Software* 4(43): 1686.

# 2. Additional Tables

Table A: Summary Results of test for Global Spatial Autocorrelation (Global Moran’s Test)

| Category | NFHS | Moran's Index | Expected Index | Variance | Z-score | p-value |
| --- | --- | --- | --- | --- | --- | --- |
| Overall | NFHS-4 | 0.7745517 | -0.001385042 | 0.0005329027 | 33.61262 | <0.0001 |
| Overall | NFHS-5 | 0.7713070 | -0.001385042 | 0.0005336518 | 33.44856 | <0.0001 |
| Public | NFHS-4 | 0.7403683 | -0.001385042 | 0.0005329649 | 32.12996 | <0.0001 |
| Public | NFHS-5 | 0.7737901 | -0.001385042 | 0.0005335750 | 33.55847 | <0.0001 |
| Private | NFHS-4 | 0.6563449 | -0.001385042 | 0.0005344827 | 28.44990 | <0.0001 |
| Private | NFHS-5 | 0.6546143 | -0.001385042 | 0.0005347949 | 28.36676 | <0.0001 |

Table B: Getis-Ord General G Statistics for spatial clustering of caesarean section deliveries

| Category | NFHS | G-Statistic | Z-score | p-value |
| --- | --- | --- | --- | --- |
| Overall | NFHS-4 | 0.01099 | 18.67214 | <0.0001 |
| Overall | NFHS-5 | 0.01081 | 19.13736 | <0.0001 |
| Public | NFHS-4 | 0.00810 | 8.28497 | <0.0001 |
| Public | NFHS-5 | 0.00808 | 9.00839 | <0.0001 |
| Private | NFHS-4 | 0.01082 | 19.18451 | <0.0001 |
| Private | NFHS-5 | 0.00749 | 17.28355 | <0.0001 |

# 3. Additional Figures

| Fig A: Distribution of the density of the proportion of c-section births across rural and urban India |
| --- |
| 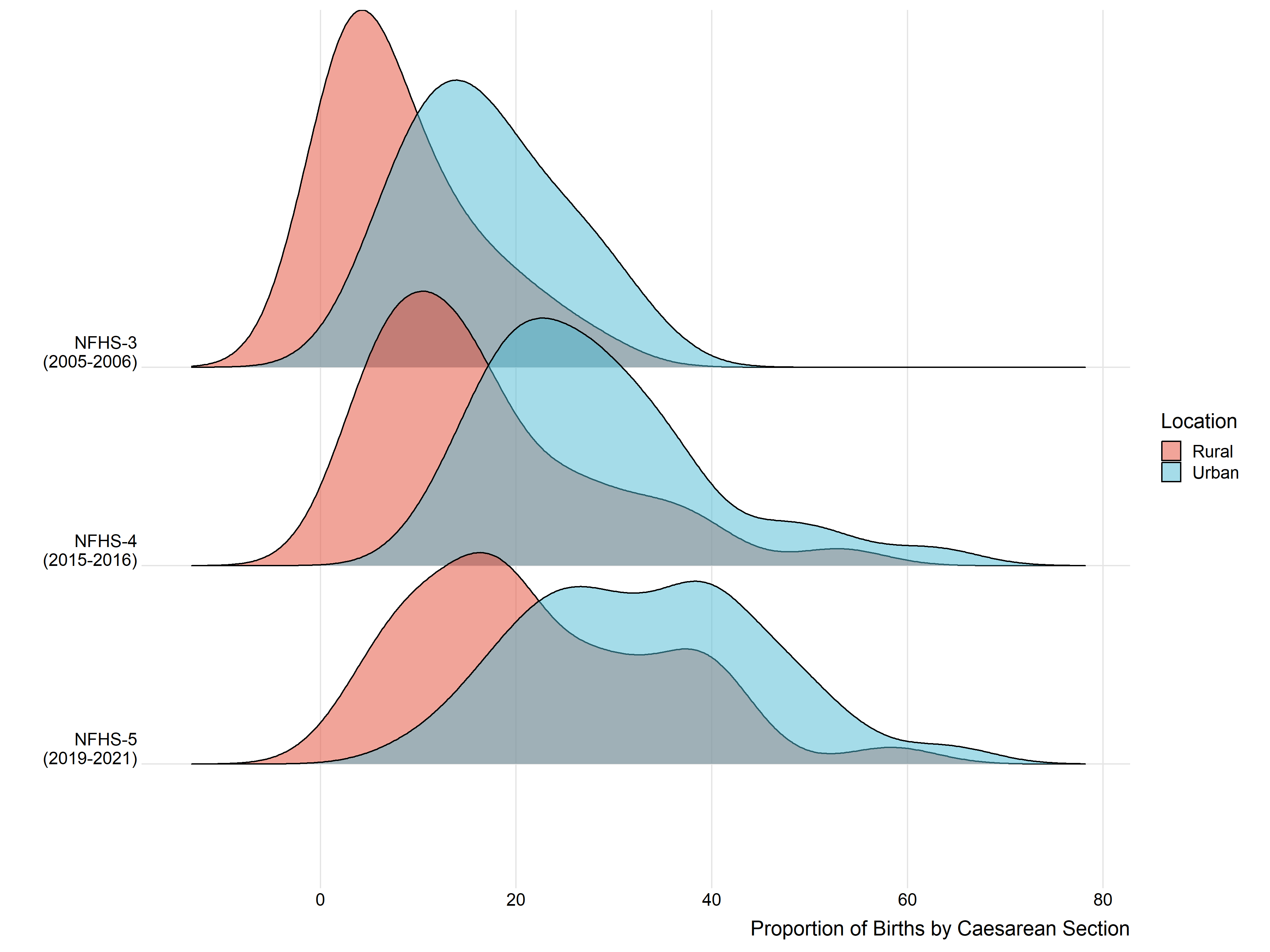  Fig B: Increase in median proportion of c-section births (%) by place of residence, place of birth and NFHS survey |
| 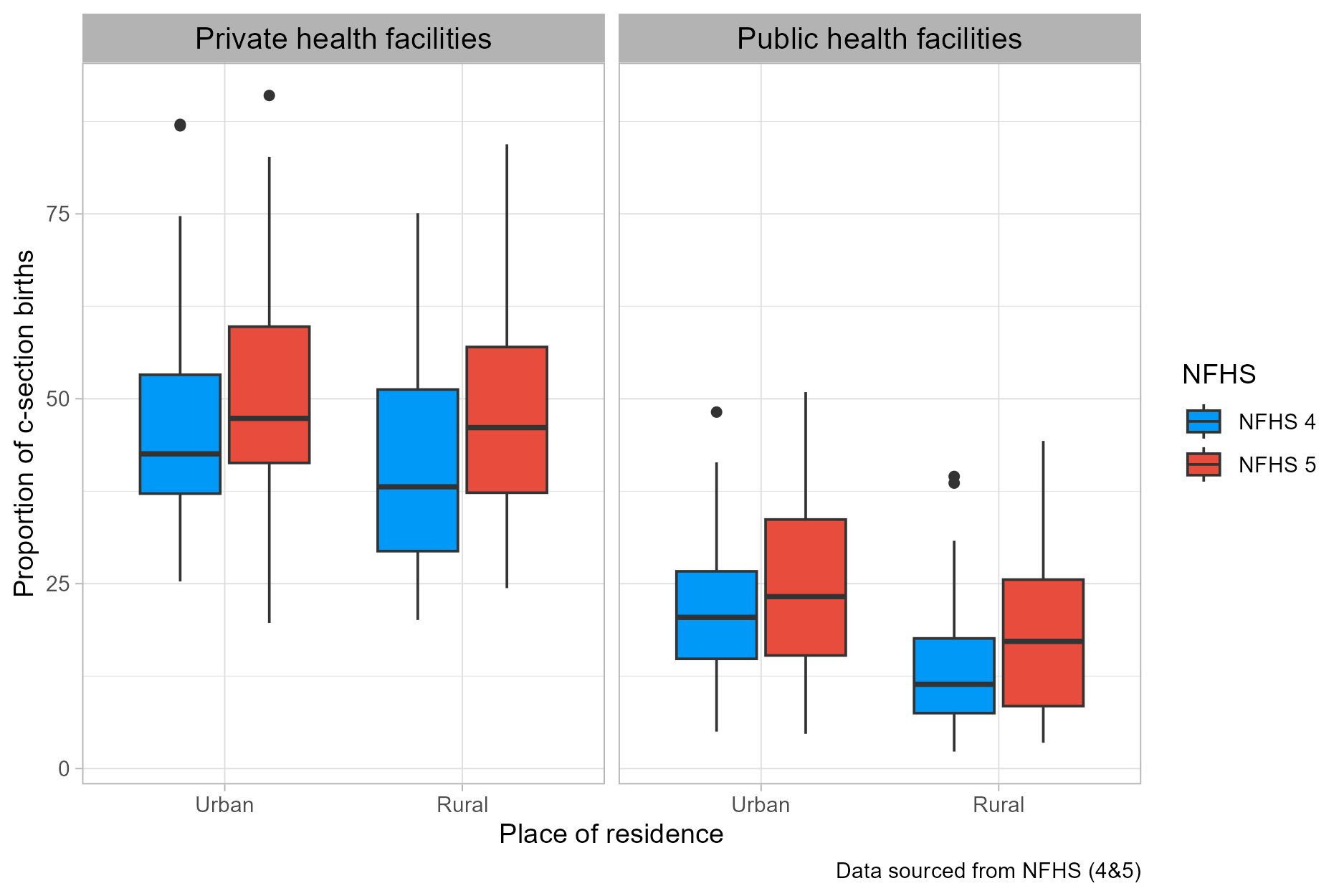 |
|  |
