# Supplementary material for: Exploring spatial clusters of caesarean sections across India - Insights from National Family Health Survey data
Source: PLOS Glob Public Health. 2026 Mar 5;6(3):e0006070. doi: 10.1371/journal.pgph.0006070 (PMC12962522; doi:10.1371/journal.pgph.0006070)
Supplement: Attachment — Submitted filename: Response_to_Reviewers.docx [file pgph.0006070.s003.docx]

Response to Reviewers

# Reviewer #1:

### Minor Typographical Errors:

line 16 clustering-----clusters
line 17 Global Moran's---- add index/statistic
line 35 delivery compared ----- delivery as compared
line 39 TThe ---- The
line 47 focus of research ---- focus of many researchers
line 57 put space after full stop in (6).In
line 98 R(?)
line 159-161 Three zones ---- NFHS 5. repeated twice
line 165 delete (6.44%) duplicated.

Response:

We apologise for the oversight and typographical errors. We have made the necessary corrections in the manuscript as suggested by the reviewer.

### Major Corrections:

| Sno | Comment | Response |
| --- | --- | --- |
| 1 | In line 159, You have mentioned that the national average C-section. Could please specify the source and does it contradict with your assumption that it is a first kind of research in India. | Thank you for your comment. We would like to clarify that we were referring to the NFHS-4 & NFHS-5 national averages of c-section as mentioned by the Government of India in their NFHS Reports. While the national averages exist, our study is the first (to the best of our knowledge) to conduct comprehensive spatial clustering analysis at the district level. |
| 2 | It would be better, if you could have made the description of the descriptive statistics short and precise instead of making duplicated similar results. | We appreciate the reviewer’s comments of making the description of the descriptive statistics more concise. We have reduced the description of the results by discussing only the most relevant findings and removing any duplicate descriptions. |
| 3 | In figure 4, at the northern tip region, the graph shows a change from Low-High to High-High spatial distribution. But when you come to figure five there is no change in both private and public hospitals. could you please figure it out. | Thank you for this important observation. We have carefully examined the spatial clustering patterns in the northern region across Figures 4 and 5. The overall clustering pattern (Figure 4) reflects the combined effect of public and private facilities. When disaggregated by facility type (Figure 5), the clustering patterns can differ because they represent distinct healthcare delivery systems with different spatial distributions.  The apparent discrepancy noted by the reviewer relates to Ladakh, which appears at the northern tip of India. This union territory (UT) presents unique challenges for our analysis:  **1. Administrative Changes:** Ladakh was carved out as a separate UT from Jammu & Kashmir only in 2019, between the two NFHS survey periods. Therefore, Ladakh data were not independently available in NFHS-4 (2015-16) and only became available as a separate administrative unit in NFHS-5 (2019-21).  **2. Data Imputation:** For NFHS-4, we imputed values for Ladakh using the Jammu & Kashmir state average, as explained in our methodology (lines 115-117). This imputation may not accurately reflect the actual delivery practices in this distinct geographic and demographic region.  **3. Healthcare Infrastructure:** Ladakh is sparsely populated with limited healthcare infrastructure. The lack of private health facilities in this region means that the facility-type breakdown in Figure 5 shows different patterns compared to the overall clustering in Figure 4, which combines both public and private facility data. |
| 4 | In figure 5, figures a and b are not labeled clearly. | Thank you for pointing it out. We have rectified it in the revised figure. |
| 5 | Tables S1 and S2 are the main results of the research but not yet discussed and concluded in detail. Hence, I would suggest including them in the main results and discuss in detail. | Thank you for the comment. We agree with your suggestion and we have included it in the main manuscript as suggested and have discussed these results in detail. |
| 6 | could you please show where is the result of the analysis of Moren's I index/statistic | This was an oversight on our side, we have now included the results of the Global Moran’s Index in the supplementary appendix (Table S1) |
| 7 | My final recommendation would be most of the contents of the discussion are literature reviews. It would be better I it is cut and paste in the introduction section. | We have done the rectifications as suggested by the reviewer. We have revised the Introduction Section to include the literature reviews and remove redundant and repeating information. We have revised the results section to present the findings of the ANOVA test. We have completely re-written the Discussion section to bring flow and clarity while also expanding the discussion to include aspects on policy implications. |

# Reviewer #1:

I consider the article suitable for publication after minor revisions focused on improving the fluency of English writing and expanding the discussion of actionable policy implications.

Response:

We have made the necessary corrections in the manuscript as suggested by the reviewer. We have completely re-written the Discussion section to bring flow and clarity while also expanding the discussion to include aspects on policy implications.
