# Supplementary material for: Exploring spatial clusters of caesarean sections across India - Insights from National Family Health Survey data
Source: PLOS Glob Public Health. 2026 Mar 5;6(3):e0006070. doi: 10.1371/journal.pgph.0006070 (PMC12962522; doi:10.1371/journal.pgph.0006070)
Supplement: Attachment — Submitted filename: Response_to_Reviewers_v2.docx [file pgph.0006070.s004.docx]

**Response to Editor Comments**

### **Minor Revision:**

Previous studies have investigated the spatial clustering of Caesarean sections in India, e.g. https://doi.org/10.1007/s13224-025-02150-4 and https://doi.org/10.1186/s12889-024-19984-8. This is relevant literature that should be cited. Additionally, please discuss the contribution of the current study in the context of this previously published research.

**Response:**

We thank the editor for bringing these important studies to our attention. We have carefully reviewed both papers and have incorporated them into our revised manuscript. Below, we describe the changes made.

Paper 1: Singh KJ et al. (2025): "Identification of Spatial Hotspots Clustering and Geographically Weighted Regression Analysis to Assess Predictors of Cesarean Section Delivery in Northeastern States, India." identified hotspots in districts of Sikkim, Tripura, Assam, and Manipur, and found maternal age, birth order, education, BMI, and wealth quintile as significant spatially varying determinants.
Paper 2: Singh M, Singh A, Gupta J. (2024): "Exploring Cesarean Section Delivery Patterns in South India: A Bayesian Multilevel and Geospatial Analysis of Population-Based Cross-Sectional Data." reported significant regional disparities and identified maternal age, education, healthcare facility type, and wealth as key determinants.

While we have already included one of the paper as a reference in our submission (Singh et al (2024)), we agree that this has been done only in the context of catastrophic health expenditures. We have now expanded its citation to appropriately contextualise it in the spatial analysis literature.

**Changes Made to the Manuscript:**
In Introduction Section: We have revised the final paragraph of the Introduction to acknowledge prior spatial analyses and to more precisely state the unique contribution of our study. The revised text now reads:

*“Previous studies show the growing disparities in access to quality maternal healthcare, as well as inequities in the distribution of these services across different geographical regions and sociocultural contexts (5,11,16,17). Recent studies have also begun to employ spatial analytical techniques to examine C-section patterns at the subnational level. While some researchers investigated C-section delivery patterns in South India using Bayesian multilevel modelling, others applied Getis-Ord Gi* hotspot analysis and multiscale geographically weighted regression to examine spatial clustering and determinants in northeastern India (18,19). However, these studies focus on specific subregions and rely on a single survey round (NFHS-5). A comprehensive, pan-India spatial analysis examining the temporal evolution of C-section clustering across both NFHS-4 and NFHS-5, with facility-type disaggregation for public and private health sectors, remains a critical gap. The present study addresses this gap by providing the most comprehensive district-level spatial analysis of C-section clustering across India, examining trends across two survey rounds and stratifying by facility type — generating evidence to inform targeted public health policy and health systems strengthening.”*

In Discussion Section: We have added a new paragraph after the paragraph on geospatial analytical techniques to contextualise our findings with these two studies. The new paragraph reads:

*“Our findings complement and extend prior spatial analyses of C-sections in India. A study done in South India reported significant regional disparities in C-section utilization, identifying clustering driven by maternal age, education, and healthcare facility type (18). Similarly, another study identified spatial hotspots of C-section rates in districts of Sikkim, Tripura, Assam, and Manipur using NFHS-5 data for the northeastern region (19). While these studies provide valuable subregional insights, the present study advances the evidence base by offering a nationwide perspective encompassing all districts across India, examining the temporal evolution of clustering from NFHS-4 to NFHS-5, and critically, disaggregating the analysis by public and private facilities. This facility-level disaggregation reveals that private facility hotspots concentrate predominantly along the eastern coast and in northeastern regions, while public facility hotspots are predominantly in southern India; a distinction not captured by previous studies. These differential patterns underscore that policy interventions to address unnecessary C-sections must be tailored to the distinct dynamics of the public and private healthcare sectors.”*

We hope the revised version is now suitable for publication in PLOS Global Public Health.
